# Wearable vibrotactile stimulation shirts and gloves for upper extremity stroke rehabilitation: A pilot randomized controlled trial

**DOI:** 10.64898/2026.07.26.26358522

**Published:** 2026-08-05

**Authors:** Walaa Ayyad, Yeongdae Kim, Nathan Odom, Mazen Al Borno

## Abstract

Objective Conduct a randomized controlled trial to investigate the safety, feasibility, and efficacy of wearable vibrotactile stimulation shirts and gloves for upper extremity stroke rehabilitation at the inpatient rehabilitation unit. The primary outcome measure was the Fugl-Meyer Assessment Upper Extremity (FMA-UE) motor score. The secondary outcome measures were the Modified Ashworth Scale (MAS) and statistics on device safety and feasibility. Exploratory outcome measures were the FMA-UE sensation, passive joint motion, and joint pain scores. Also, the MAS for the fingers, wrist, and elbow scores. Methods A total of 24 ischemic stroke patients were recruited for this study (45 to 85 years old) during their stay at the inpatient rehabilitation unit, which averaged 15.3 ± 4.5 days. Patients were randomly assigned to a treatment or control group, with 12 participants in each group. The control group received conventional therapy only, while the treatment group received both conventional therapy and vibrotactile stimulation. Participants in the treatment group wore the vibrotactile stimulation gloves and shirts for 1.5 hours per day, 5 days per week. Upper extremity impairment and spasticity were assessed with the FMA-UE and MAS at both admission and discharge from the rehabilitation unit. In addition, feedback from patients in the treatment group was collected through a questionnaire to evaluate comfort, usability, and satisfaction. The trial was prospectively registered at ClinicalTrials.gov ( NCT06244719 ). Results The vibrotactile stimulation was safe and well-tolerated by stroke patients. No statistically significant differences were noted in FMA-UE motor scores and total MAS scores between the groups; however, exploratory analyses revealed a significant improvement in FMA-UE joint pain scores in the treatment group. A trend towards reduced wrist spasticity was observed in the treatment group, but this effect did not remain significant after correcting for multiple comparisons. Responder analyses showed a greater proportion of responders in the treatment group for both FMA-UE and MAS outcomes. Conclusions Our results show promise for a larger sample size study and follow-up work with longer durations of vibrotactile stimulation with gloves and shirts for upper extremity stroke rehabilitation. Although no significant improvements were observed in FMA-UE motor function or total MAS scores, vibrotactile stimulation was associated with reduced joint pain, potential benefits for distal spastic hypertonia, and increased responder rates.

## Full Text Availability

The license terms selected by the author(s) for this preprint version do not permit archiving in PMC. The full text is available from the preprint server.

